# Isolation of Diverse Simian Arteriviruses Causing Hemorrhagic Disease

**DOI:** 10.3201/eid3004.231457

**Published:** 2024-04

**Authors:** Teressa M. Shaw, Samuel T. Dettle, Andres Mejia, Jennifer M. Hayes, Heather A. Simmons, Puja Basu, Jens H. Kuhn, Mitchell D. Ramuta, Cody J. Warren, Peter B. Jahrling, David H. O’Connor, Liupei Huang, Misbah Zaeem, Jiwon Seo, Igor I. Slukvin, Matthew E. Brown, Adam L. Bailey

**Affiliations:** University of Wisconsin, Madison, Wisconsin, USA (T.M. Shaw, S.T. Dettle, A. Mejia, M.D. Ramuta, D.H. O’Connor, L. Huang, M. Zaeem, J. Seo, I.I. Slukvin, M.E. Brown, A.L. Bailey);; Wisconsin National Primate Research Center, Madison (S.T. Dettle, A. Mejia, J.M. Hayes, H.A. Simmons, P. Basu, D.H. O’Connor, I.I. Slukvin);; National Institutes of Health, Fort Detrick, Frederick, Maryland, USA (J.H. Kuhn, P.B. Jahrling);; The Ohio State University, Columbus, Ohio, USA (C.J. Warren)

**Keywords:** arterivirid, *Arteriviridae*, arterivirus, engraft, induced pluripotent stem cell, iPSC, Kibale red colobus virus, KRCV-1, NHP, nonhuman primate, PBJV, Pebjah virus, SHFV, simarterivirin, Simarterivirinae, simarterivirus, simian hemorrhagic fever virus, southwest baboon virus, SWBV, viruses, zoonoses, United States

## Abstract

Genetically diverse simian arteriviruses (simarteriviruses) naturally infect geographically and phylogenetically diverse monkeys, and cross-species transmission and emergence are of considerable concern. Characterization of most simarteriviruses beyond sequence analysis has not been possible because the viruses fail to propagate in the laboratory. We attempted to isolate 4 simarteriviruses, Kibale red colobus virus 1, Pebjah virus, simian hemorrhagic fever virus, and Southwest baboon virus 1, by inoculating an immortalized grivet cell line (known to replicate simian hemorrhagic fever virus), primary macaque cells, macrophages derived from macaque induced pluripotent stem cells, and mice engrafted with macaque CD34+-enriched hematopoietic stem cells. The combined effort resulted in successful virus isolation; however, no single approach was successful for all 4 simarteriviruses. We describe several approaches that might be used to isolate additional simarteriviruses for phenotypic characterization. Our results will expedite laboratory studies of simarteriviruses to elucidate virus-host interactions, assess zoonotic risk, and develop medical countermeasures.

Simarteriviruses (order Nidovirales, family Arteriviridae, subfamily Simarterivirinae) are genetically diverse viruses that naturally infect cercopithecoid monkeys throughout sub-Saharan Africa. Many divergent simarteriviruses can infect macaques, although severity of disease ranges from subclinical to highly fatal in this nonnatural host (*1*,*2*). Simarteriviruses are not known to infect humans, but the close evolutionary history of simarterivirus hosts and humans, their interhost and intrahost diversity, and various molecular characteristics suggest that those viruses might pose a zoonotic threat (*3*–*5*).

Simian hemorrhagic fever virus (SHFV), the best characterized simarterivirus, was identified as the cause of a highly fatal hemorrhagic fever epizootic at a National Institutes of Health quarantine facility in Bethesda, Maryland, USA, in 1964, affecting captive rhesus monkeys from Asia (*Macaca mulatta*), crab-eating macaques (*Macaca fascicularis*), and stump-tailed macaques (*Macaca arctoides*) (*6*–*8*). During the next 3 decades, several other epizootics in captive macaques were attributed to SHFV, but retrospective sequencing analyses indicated that other highly diverse simarteriviruses caused some of those outbreaks despite similar clinical manifestations (*9*). Healthy monkeys from Africa housed adjacent to the affected macaques from Asia were long suspected to be the natural simarterivirus hosts, but simarterivirus identification in healthy wild monkeys from Africa only began in 2011, when metagenomic sequencing was used (*10*–*14*).

Arteriviruses in general, and simarteriviruses in particular, are difficult to isolate. SHFV will only replicate to high titers in the grivet-derived cell line MA-104 and its subclones (e.g., MARC-145) (*5*,*8*,*15*–*19*). Although Kibale red colobus virus 1 (KRCV-1) can replicate in MARC-145 cells, replication is inconsistent, and virus production is low. Thus, immortalized cell lines capable of sustaining robust replication of simarteriviruses other than SHFV do not exist. If a novel simarterivirus were to emerge, threatening human or animal health, the lack of in vitro systems for virus propagation and isolation would be a major hurdle in understanding basic virology and developing medical countermeasures. We describe a combination of approaches resulting in successful isolation of multiple diverse simarteriviruses.

## Methods

### Biosafety and Ethics Statement

We obtained primary macaque tissues from the Nonhuman Primate Biologic Materials Distribution Core service at the Wisconsin National Primate Research Center in Madison, WI, USA. All experiments involving simarteriviruses were performed in a Biosafety Level 3 laboratory by trained personnel equipped with powered air purifying respirators, according to approved standard operating procedures. All experiments were approved before initiation by the institutional Biosafety Committee and Animal Care and Use Committee at the University of Wisconsin–Madison. 

### Virus Choices

Most simarteriviruses are uncultivable in vitro. Viruses that have been successfully used for in vivo studies are KRCV-1, SHFV, and Southwest baboon virus 1 (SWBV-1); those infections were performed in cercopithecoid nonhuman primates (NHPs). Small rodent models of simarterivirus infection would be useful to further characterize simarterivirus transmission and pathogenesis as well as to develop medical countermeasures against simarteriviruses of zoonotic concern. We chose the viruses evaluated in this study because they were previously discovered in the laboratory of David H. O’Connor at the University of Wisconsin–Madison by using unbiased deep sequencing, and we had retained residual source material from those studies. Pebjah virus (PBJV) had not been isolated, and our source material was not known to contain infectious virus. SWBV-1 had not been isolated, but we had reason to believe that our source material contained infectious virus because we had been able to productively infect NHPs with this material, as previously described (Table) (*25*).

**Table:** Viruses used in study of isolation of diverse simarteriviruses causing hemorrhagic disease*

Simarterivirus	Year of discovery	Place of discovery	Natural monkey host from Africa	Disease in macaques from Asia	References
Kibale red colobus virus 1	2011	Kibale National Park, Uganda	Ugandan red colobus (*Piliocolobus tephrosceles*)	Mild (experimental exposure of crab-eating macaques)	(*10*,*17*)
Pebjah virus	1989, 2015	Primate Research Institute of New Mexico State University, Alamogordo, New Mexico, USA	Unknown	Severe/lethal (epizootic among captive crab-eating macaques)	(*9*,*20*,*21*)
Simian hemorrhagic fever virus	1964	Primate Quarantine Unit at the National Institutes of Health, Bethesda, Maryland, USA	Possible: Olive baboons (*Papio anubis*), patas monkeys (*Erythrocebus patas*)	Severe/lethal (epizootic among captive crab-eating macaques, rhesus monkeys, and stump-tailed macaques; experimental exposure of crab-eating macaques, Japanese macaques; rhesus monkeys, and stump-tailed macaques)	(*6*–*8*,*17*,*22*–*24*)
Southwest baboon virus 1	2014	Southwest National Primate Research Center, San Antonio, Texas, USA	Olive baboons (*Papio anubis*)	Subclinical (experimental exposure of rhesus monkeys)	(*12*,*22*,*25*)
*NA, not applicable.					

### Source Material

Low-titer MARC-145 cell culture supernatant containing KRCV-1 (*10*,*13*) was provided by Cody Warren (The Ohio State University). We obtained olive baboon plasma containing SWBV-1 RNA, cell culture supernatants containing SHFV or recombinant SHFV expressing enhanced green fluorescent protein (eGFP), and material containing PBJV RNA from previous studies (*9*,*12*,*15*,*17*,*25*). For in vitro experiments, we provide the multiplicity of infection (MOI) for SHFV in PFU, determined by plaque assay (*16*). We were unable to make similar MOI estimations for KRCV-1, PBJV, and SWBV-1 because we lacked a method for assessing virus titers; therefore, we used the same volumes of starting material for each of those viruses.

### Quantification

For quantification of virus RNA in cell culture supernatants or serum samples, we extracted RNA from 20 or 50 μL of samples by using a KingFisher Flex System and MagMAX Viral and Pathogen Nucleic Acid Isolation Kit (both ThermoFisher Scientific, https://www.thermofisher.com). We eluted RNA in 50 μL water and used 8.5 μL RNA for quantitative reverse transcription PCR (qRT-PCR). We designed virus-specific TaqMan assays to detect each virus by using simarterivirus genome sequences from GenBank. We used Primer3 (https://primer3.org) for primer and probe design, and Integrated DNA Technologies (https://idtdna.com) synthesized the primers and probes according to the FAM and Zen/Iowa-Black dual-quencher system. Oligonucleotide sequences were: KRCV-1-F, 5′-ACACGGCTACCCTTACTCC-3′; KRCV-1-R, 5′-TCGAGGTTAARCGGTTGAGA-3′; KRCV-1-P, 5′-TTCTGGTCCTCTTGCGAAGGC-3′; PBJV-F, 5′-GAGGATGGTCGCCTCAACTA-3′; PBJV-R, 5′-AAGGACCCTCGTCAAATTCA-3′; PBJV-P, 5′-TGCTGTCATCACACCAGATG-3′; SHFV-F, 5′-CGACCTCCGAGTTGTTCTACCT-3′; SHFV-R, 5′-GCCTCCGTTGTCGTAGTACCT-3′; SHFV-P, 5′-CCCACCTCAGCACACATCAAACAGCT-3′; SWBV-1-F, 5′-GCTTGCTGGTAAGATTGCCA-3′; SWBV-1-R, 5′-GTCCTAGGAGCAGCTGTTGG-3′; and SWBV-1-P, 5′-TGATTAACCTGAGGAAGTATGGCTGGC-3′. We used the TaqMan RNA-to-C_T_ 1-Step Kit (ThermoFisher Scientific) for qRT-PCR, which we performed on a Quantstudio 6 Pro thermocycler (ThermoFisher Scientific) as follows: reverse transcription at 48°C for 15 minutes, initial denaturation at 95°C for 10 minutes, a subsequent 50 cycles at 95°C for 15 seconds, and then incubation at 60°C for 1 minute, during which signal acquisition was performed. Using a custom-designed, in vitro-transcribed RNA standard curve for SHFV, we determined that a cycle threshold (Ct) of ≈38–40 corresponded to ≈10 RNA copies/reaction and, thus, was a generalizable limit of detection in this study. We extrapolated the standard curve to a rough approximation of the copies/mL of the SHFV *N* gene, which encodes nucleoprotein: a Ct of 29 was ≈10^6^ copies/mL, Ct of 22 was ≈10^8^ copies/mL, and Ct of 15 was ≈10^10^ copies/mL.

### MA-104 Cell Culture

MA-104 cells, derived from grivet (*Chlorocebus aethiops*) embryonic kidneys, were provided by Siyuan Ding (Washington University, St. Louis, MO, USA). We cultured the cells in Dulbecco Modified Eagle Medium containing 1% HEPES buffer, 1% sodium pyruvate, and 1% L-glutamine (GIBCO/ThermoFisher Scientific), and 10% heat-inactivated fetal calf serum (FCS; Omega Scientific, https://www.omegascientific.com).

### Rhesus Monkey Peripheral Blood Mononuclear Cells 

We obtained ≈15 mL of rhesus monkey whole blood in EDTA and added 15 mL of Roswell Park Memorial Institute (RPMI) 1640 medium (ThermoFisher Scientific) containing 10% FCS. We overlayed the diluted blood with 15 mL Ficoll-Paque (Cytiva, https://www.cytivalifesciences.com) and centrifuged at 800 × *g* for 30 minutes without braking. We extracted the buffy coat and treated the cells once with ACK Lysing Buffer (GIBCO/ThermoFisher Scientific), according to the manufacturer’s instructions. We washed the cells 2 times with RPMI 1640 medium and then plated them for differentiation, achieved by adding bulk peripheral blood nononuclear cells (PBMCs) to flasks containing minimum essential medium α (MEM α; GIBCO/ThermoFisher Scientific), 20% heat-inactivated FCS, 1% type AB human serum (Sigma Aldrich, https://www.sigmaaldrich.com), 50 µmol/L 2-mercaptoethanol (GIBCO/ThermoFisher Scientific), 20 µg/mL human macrophage colony-stimulating factor (M-CSF; Peprotech, https://www.peprotech.com), 10 µg/mL human interleukin-1 beta (IL-1β; Peprotech), and antimicrobial/antifungal solution containing penicillin, streptomycin, and amphotericin B (GIBCO/ThermoFisher Scientific). We allowed the cells to differentiate for 6 days, changing medium every 2 days. We removed differentiated cells by using Cellstripper (Corning, https://www.corning.com) according to the manufacturer’s recommendations and then plated the cells for experiments.

We inoculated cells with an SHFV MOI of 0.1 or equivalent volumes of KRCV-1, PBJV, or SWBV-1 in MEM α medium containing 2% FCS and incubated for 1 h at 37°C, gently rocking every ≈15 min. We washed monolayers 2 times with phosphate-buffered saline (PBS) and then added complete growth medium. We collected cell supernatants at different timepoints and stored them at −80°C before RNA extraction.

### Rhesus Monkey Splenocytes

We obtained spleen samples from rhesus monkeys during necropsies of animals used for other studies. We mechanically dissociated 2–10 g of tissue by using scissors and a scalpel in ≈10 mL of RPMI 1640 medium containing heat-inactivated 10% FCS and antimicrobial/antimycotic solution and then passed the tissue through a 100-µm filter to obtain a single cell suspension. We treated the cells with ACK Lysing Buffer and washed 2 times with ≈20 mL RPMI medium before plating for experiments or inducing differentiation. We let bulk splenocytes adhere to tissue culture dishes for 12 hours and added nonadherent cells to a separate flask with differentiation medium prewarmed to 37°C. We maintained cells under standard tissue culture conditions (5% CO_2_, 37°C) in differentiation medium for 6 days before performing experiments.

We inoculated splenocyte-derived macrophages with an SHFV MOI of 0.1 or equivalent volumes of KRCV-1, PBJV, or SWBV-1 in MEM α containing 2% FCS for 1 hour. We washed the monolayers before adding complete growth medium and stored cell supernatants at −80°C before RNA extraction.

### Flow Cytometry of Rhesus Monkey Splenocytes

We obtained a single cell suspension of rhesus monkey splenocytes and treated them with ACK Lysing Buffer as previously described. After treatment, we centrifuged the cells for 10 minutes at 300 × *g*, resuspended them in labeling buffer (PBS containing 0.5% bovine serum albumin and 2 mmol/L EDTA), and then incubated with NHP CD11b microbeads (Miltenyi Biotec, https://www.miltenyibiotec.com) for 15 min at 4°C. We washed the cells 2 times with PBS containing 0.5% bovine serum albumin and 2 mmol/L EDTA and then resuspended in 500 µL of the same buffer. We attached LS columns to a MACS manual magnetic cell separator (both Miltenyi Biotec), prepared them according to manufacturer’s instructions, and added microbead-labeled cell suspensions to the columns and let unlabeled cells flow through. We washed the LS columns 3 times with buffer and, after collecting unlabeled cells, we removed the columns from the MACS separator and placed them into 15-mL conical tubes. We added 5 mL buffer to each column, recovered CD11b-enriched cells, and added differentiation medium to the labeled cells, replenishing the medium every 2 days.

Six days after initiating differentiation, we inoculated splenocyte-derived macrophages with SHFV-eGFP at an MOI of 0.1 without washing. After 24 hours, we harvested the cells by using Cellstripper and transferred them to a nontissue culture-treated 96-well V-bottom plate (Corning), which we centrifuged for 5 minutes at 500 × *g*. We rinsed the cells 2 times in fluorescence-activated cell sorter (FACS) buffer (PBS, 2% FCS, 1 mmol/L EDTA [Invitrogen/ThermoFisher Scientific]) before adding ViaDye (Cytek Biosciences, https://www.cytekbio.com) to the cells according to the manufacturer’s instructions. After rinsing the cells 2 times in FACS buffer, we added Human TruStain FcX Block (Biolegend, https;//www.biolegend.com), incubated for 10 minutes in the dark, then added human CD14 antibody (Biolegend) and incubated for an additional 20 minutes in the dark. After incubation, we washed the cells 2 times in FACS buffer before fixing for 20 minutes in 4% paraformaldehyde (Electron Microscopy Sciences, https://www.emsdiasum.com) diluted in PBS and then rinsed the cells 2 times in with FACS buffer and stored at 4°C before flow cytometric analysis on an Attune Flow Cytometer (ThermoFisher Scientific). We performed analysis by using FlowJo version 10.8 software (BD Biosciences, https://www.bdbiosciences.com).

### Macrophages Derived from Induced Pluripotent Stem Cells

We generated induced pluripotent stem cells (iPSCs) from crab-eating macaques as previously described (*26**–**29*). In brief, we isolated fibroblasts from skin punches, reprogrammed them by using episomally-encoded Yamanaka factors, and maintained an undifferentiated state by passaging the cells on mouse embryonic fibroblasts every 3 days. We generated multipotent hematopoietic progenitors (MHPs) from iPSCs as described previously (*26*,*27*). In brief, we cultured iPSC aggregates on OP9 feeder cell layers for 10 days in NHP medium (MEM α, 10% Hyclone fetal bovine serum [Cytiva] that was not heat inactivated, 50 µmol/L 2-mercaptoethanol), and day-specific amounts of cytokines and chemicals (Peprotech). At differentiation day 10, we collected floating MHPs and either froze the cells in 10% vol/vol dimethyl sulfoxide/90% fetal bovine serum or used them to generate macrophages. We further differentiated MHPs to macrophages by incubating them in ultralow-attachment 6-well plates (Corning) for 6 days in NHP medium containing 20 ng/mL of M-CSF and 10 ng/mL of IL-1β (*28*). Every 2 days, we added 2 mL NHP medium/well and additional cytokines to maintain final concentrations of 20 ng/mL M-CSF and 10 ng/mL IL-1β. We maintained the iPSC-derived macrophages in fresh NHP medium containing M-CSF and IL-1β and performed complete medium changes and replating every 6 days.

For virus exposure experiments, we plated cells ≈2.5 × 10^5^ cells/well in 6-well ultralow attachment plates and incubated with inoculum for 1 hours at 37°C and 5% CO_2_, gently rocking the plates every ≈15 min. We removed the inoculum, washed the cells 3 times with PBS, and then replenished with prewarmed medium.

### Mice Engrafted with Macaque Immune Cells 

Mice were prepared as previously described (*29*). In brief, we created engrafted mice by using fetal (≈100 days’ gestation) tissue from rhesus monkeys obtained from the Wisconsin National Primate Research Center. We used immunodeficient NSG-SGM3 (NOD.Cg-*Prkdc^scid^* *Il2rg^tm1Wjl^* Tg[CMV-IL3,CSF2,KITLG]1Eav/MloySzJ) and NOG-EXL (NOD.Cg-*Prkdc^scid^* *Il2rg^tm1Sug^* Tg[SV40/HTLV-IL3,CSF2]10–7Jic/JicTac) transgenic mice (both from Taconic Biosciences, https://www.taconic.com) as hosts for the experiments and unengrafted NSG-SGM3 transgenic mice and background-matched wildtype NOR/LtJ mice as controls (The Jackson Laboratory, https://www.jax.org). Before the operation, we myeloablated the transgenic mice by using busulfan (*30*). We engrafted the mice surgically with 1–2 primate thymus fragments (≈1 mm^3^) placed under the kidney capsule and injected 8 × 10^4^ to 2 × 10^5^ primate liver hematopoietic stem/progenitor cells through the tail vein; we treated the mice with 2 doses (days 0 and 7 after surgery) of 100 µg CD2 antibody via retro-orbital injection. We collected and analyzed blood samples 6–7 weeks after surgery from all mice to assess primate immune cell engraftment (NHP CD45+ cells). 

### Virus Exposure Studies in Mice

We analyzed 5 cohorts of laboratory mice and recorded specific genetic background, engraftment status, and virus exposure for each cohort (Appendix Table). After engraftment and verification of graft health via flow cytometry (*29*), we transferred the mice to an animal Biosafety Level 3 laboratory and acclimated them for 4–8 days. Before virus exposure, the mice were sedated by using isoflurane gas, after which we intravenously inoculated 50 μL of virus-positive material into the retro-orbital space by using a 31-gauge needle and injected 100 μL of virus-positive material into the peritoneal cavity. We examined the mice daily and compared them with the unengrafted NSG-SGM3 and background-matched wildtype NOR/LtJ controls. We collected ≈5 drops of blood via submandibular venipuncture at 2, 6, and 12 days after virus exposure for RNA extraction and virus load measurements.

## Results

### Simarteriviruses in Grivet MA-104 Cells

**Figure 1:**
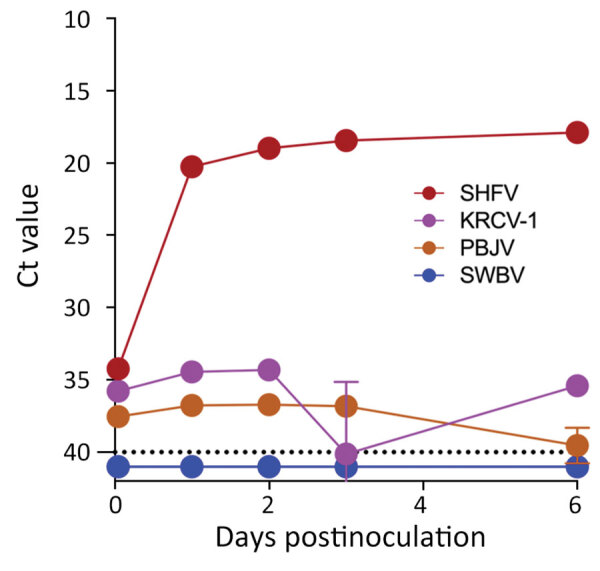
Simarterivirus infection of grivet embryonic kidney cell line in study of isolation of diverse simarteriviruses causing hemorrhagic disease. MA-104 cells were inoculated with SHFV at a multiplicity of infection of 1 and inoculated with KRCV-1, PBJV, or SWBV-1 by using sample volumes equivalent to that of SHFV (n = 3 experiments for each virus). Supernatants were collected at the indicated timepoints and quantitative reverse transcription PCR was used to measure simarterivirus nucleoprotein gene levels. Dashed line indicates limit of detection. Only SHFV replicated in grivet MA-104 cells. Ct, cycle threshold; KRCV-1, Kibale red colobus monkey virus 1; PBJV, Pebjah virus; SHFV, simian hemorrhagic fever virus; SWBV-1, Southwest baboon virus 1.

The grivet embryonic kidney cell line, MA-104 (including several subclones, such as MARC-145), is susceptible to infection by many divergent arteriviruses. However, with the exception of SHFV, MA-104 cells do not support robust infection with other simarteriviruses (*5*,*8*,*15*–*19*). To confirm this lack of susceptibility, we exposed MA-104 cells to residual monkey serum and other biospecimens containing KRCV-1, PBJV, SHFV, or SWBV-1. To evaluate productive infection by those viruses, we monitored cultures during <21 days for cytopathic effect (CPE) and used qRT-PCR targeting the virus-specific *N* gene to detect increasing concentrations of *N* RNA in cell culture supernatant; we made partial growth medium changes as needed. As expected, MA-104 cultures inoculated with SHFV displayed rapid CPE accompanied by an increase in SHFV *N* RNA levels. However, MA-104 cells inoculated with KRCV-1, PBJV, or SWBV-1 maintained normal morphologic characteristics and did not display increases in virus *N* RNA levels (Figure 1). Our results confirmed that MA-104 cells cannot be used to grow or isolate SWBV-1, KRCV-1, or PBJV.

### Simarterivirus Isolation from Primary Rhesus Monkey Macrophages and Splenocytes

**Figure 2:**
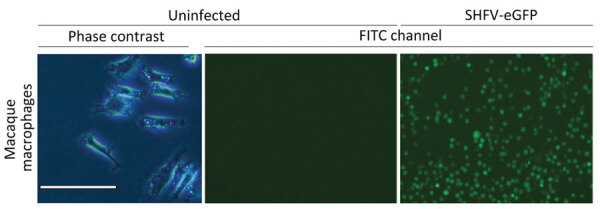
Primary rhesus monkey macrophages infected with simian hemorrhagic fever virus in study of diverse simarteriviruses causing hemorrhagic disease. Macrophages were isolated from spleen tissue and mock infected or infected with SHFV-eGFP. Left panel shows isolated macrophages; scale bar indicates 120 μm. Middle panel shows cells 24 hours after mock infection and right panel shows cells 24 hours after infection with SHFV-eGFP at a multiplicity of infection of 0.1; original magnification ×40. SHFV-eGFP, simian hemorrhagic fever virus–enhanced green fluorescent protein.

**Figure 3:**
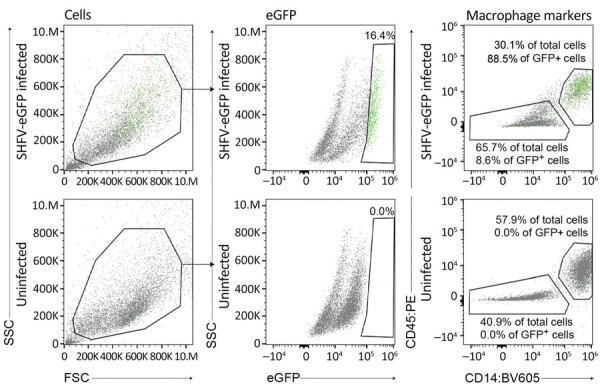
Flow cytometry of SHFV-eGFP–infected (top plots) and uninfected (bottom plots) splenocytes in study of isolation of diverse simarteriviruses causing hemorrhagic disease. Green dots indicate cells infected with SHFV-eGFP. BV605, brilliant violet 605 dye; eGFP, enhanced GFP; FSC, forward scatter; GFP, green fluorescent protein; PE, phycoerythrin; SHFV, simian hemorrhagic fever virus; SSC, side scatter.

**Figure 4:**
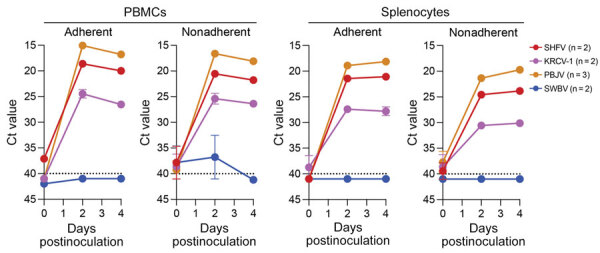
Ct values for virus-specific nucleoprotein RNA from rhesus monkey cells in study of diverse simarteriviruses causing hemorrhagic disease. PBMCs and splenocytes were infected with SHFV at a multiplicity of infection of 0.1 and infected with KRCV-1, PBJV, or SWBV-1 by using volumes equivalent to that of SHFV. Nucleoprotein RNA was measured by using quantitative reverse transcription PCR at different times after inoculation. Dotted lines indicate limit of detection. Numbers in parentheses indicate number of experiments performed for each virus. Error bars indicate SEMs. Ct, cycle threshold; KRCV-1, Kibale red colobus monkey virus 1; PBJV, Pebjah virus; PBMCs, peripheral blood mononuclear cells; SHFV, simian hemorrhagic fever virus; SWBV-1, Southwest baboon virus 1.

**Figure 5:**
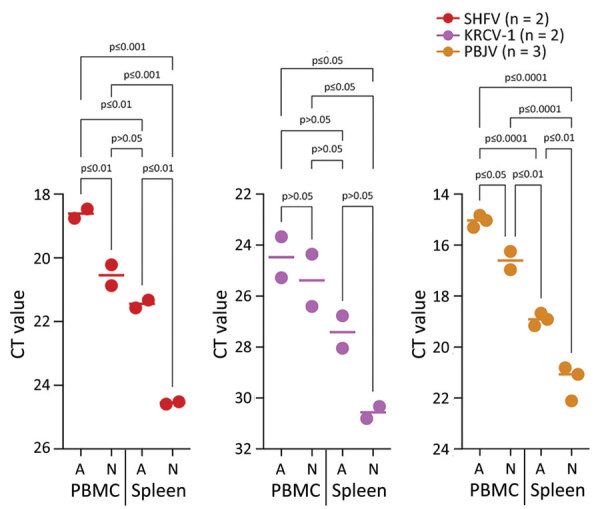
Comparisons of Ct values for different viruses infecting cells from rhesus monkeys in study of diverse simarteriviruses causing hemorrhagic disease. PBMCs and splenocytes were isolated from rhesus monkeys, infected with different simarteriviruses, and analyzed for infection by using quantitative reverse transcription PCR. Dots for each cell type and numbers in parentheses indicate number of experiments performed for each virus. Horizontal lines between dots indicate mean Ct values for each group. Statistical significance was determined by using 1-way analysis of variance. A, adherent; Ct, cycle threshold; KRCV-1, Kibale red colobus monkey virus 1; N, nonadherent; PBJV, Pebjah virus; PBMCs, peripheral blood mononuclear cells; SHFV, simian hemorrhagic fever virus.

SHFV tropism is primarily restricted to macrophages and myeloid dendritic cells in vivo, and the virus can be propagated on primary rhesus monkey macrophages (*18*,*23*,*31*,*32*). For a second attempt to isolate KRCV-1, PBJV, SWBV-1, we obtained rhesus monkey PBMCs and splenocytes. Because of the small and variable sizes of PBMCs, we were unsure whether CPE would be discernible. Therefore, we used recombinant SHFV-eGFP (*15*) as a positive control. As expected, macrophages and splenocytes became eGFP positive within 24 h after SHFV-eGFP exposure (Figures 2, 3). Next, we exposed adherent and nonadherent PBMCs and splenocytes to wild-type SHFV or samples containing the other 3 viruses. Measuring virus-specific *N* RNA over time by qRT-PCR revealed productive infections by KRCV-1, PBJV, and SHFV, but not SWBV-1 (Figures 4, 5). We showed PBJV could be isolated in cell culture, and adherent NHP PBMCs, likely monocytes, can be used to isolate previously uncultivable simarteriviruses.

### Simarterivirus Isolation from Macaque iPSCs

**Figure 6:**
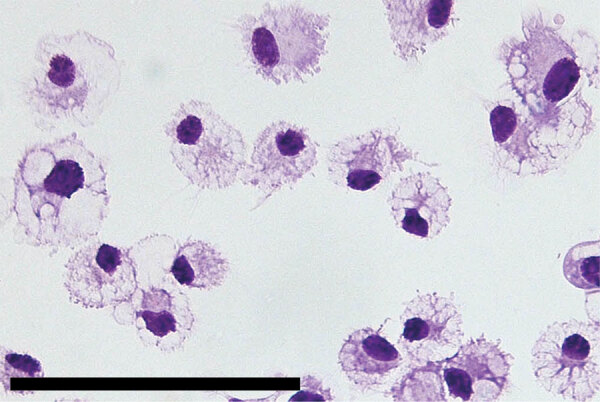
Induced pluripotent stem cells isolated from crab-eating macaques in study of diverse simarteriviruses causing hemorrhagic disease. Brightfield photograph shows induced pluripotent stem cell–derived macrophages stained with Wright-Giemsa dye. Scale bar indicates 100 μm.

**Figure 7:**
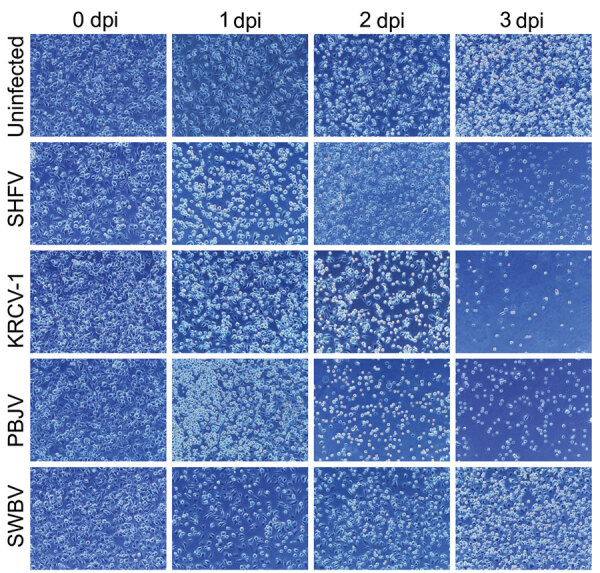
Cytopathic effect after infection of induced pluripotent stem cell–derived macrophages in study of diverse simarteriviruses causing hemorrhagic disease. Induced pluripotent stem cells from crab-eating macaques were differentiated into macrophages and mock infected (uninfected) or infected with different simarteriviruses. Cytopathic effect was monitored for 0–3 dpi. Original magnification ×100. dpi, days after inoculation; KRCV-1, Kibale red colobus monkey virus 1; PBJV, Pebjah virus; SHFV, simian hemorrhagic fever virus; SWBV-1, Southwest baboon virus 1.

**Figure 8:**
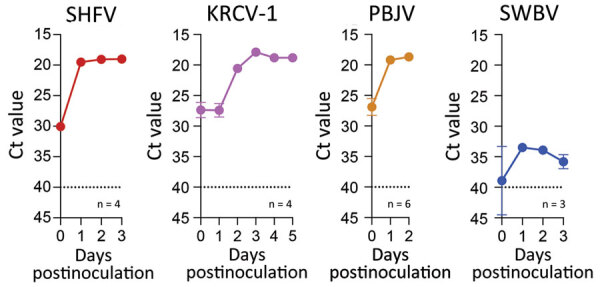
Ct values for virus-specific nucleoprotein RNA in macrophages infected with diverse simarteriviruses causing hemorrhagic disease. Induced pluripotent stem cell–derived macrophages from crab-eating macaques were infected with different simarteriviruses. Virus-specific nucleoprotein RNA was measured by using quantitative reverse transcription PCR on different days after inoculation. Dotted lines indicate limit of detection. Error bars indicate SEMs. Ct, cycle threshold; dpi, days after inoculation; iPSC, induced pluripotent stem cell; KRCV-1, Kibale red colobus monkey virus 1; LoD, limit of detection; PBJV, Pebjah virus; SHFV, simian hemorrhagic fever virus; SWBV-1, Southwest baboon virus 1.

Phylogenetically diverse cercopithecoid monkeys are susceptible to infection by various simarteriviruses (*3*); using primary macaque macrophages likely increases the chances of isolating simarteriviruses (Figure 2). However, the increasingly limited availability of macaques for biomedical research, infrastructure required for their maintenance, expertise and safety measures required to collect tissue samples, relatively small number of cells that can be purified from a tissue sample, and growing ethical concerns regarding large NHP research in general (*33*) are substantial barriers to using primary macaque macrophages as a sustainable system for simarterivirus isolation. To overcome those potential barriers, we hypothesized that macaque iPSC-derived macrophages could be used as a reproducible and sustainable system for propagating simarteriviruses. We first differentiated iPSCs from reprogrammed crab-eating macaque fibroblasts (*26*) into CD34+ MHPs, then further differentiated the MHPs into macrophages (*28*) (Figure 6). Inoculation of the macrophages with all 4 viruses revealed productive infections by KRCV-1, PBJV, and SHFV, but not SWBV-1, measured by annihilation of cells in culture (Figure 7) and virus-specific qRT-PCR (Figure 8).

### Isolation of Simarteriviruses In Vivo 

**Figure 9:**
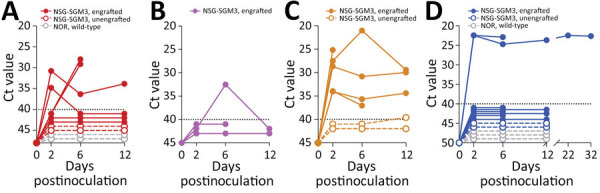
Ct values for virus-specific nucleoprotein RNA in mice engrafted with rhesus monkey immune cells infected with diverse simarteriviruses causing hemorrhagic disease. NSG-SGM3 (NOD.Cg-*Prkdc^scid^* *Il2rg^tm1Wjl^* Tg[CMV-IL3,CSF2,KITLG]1Eav/MloySzJ) mice were engrafted with fetal CD34+-enriched hematopoietic stem and progenitor cells isolated from rhesus monkeys. Engrafted and unengrafted NSG-SGM3 transgenic mice and background-matched wild-type NOR/LtJ mice were injected intraperitoneally and intravenously with 1 of 4 viruses: A) simian hemorrhagic fever virus; B) Kibale red colobus monkey virus 1; C) Pebjah virus; or D) Southwest baboon virus 1. Production of virus-specific nucleoprotein RNA in blood was measured over time by using quantitative reverse transcription PCR. Dotted lines indicates limit of detection. Ct, cycle threshold.

We hypothesized that immunodeficient mice engrafted with fetal rhesus monkey thymus- and liver-derived CD34+-enriched hematopoietic stem and progenitor cells (*29*) would be susceptible to simarterivirus infection. Mice were inoculated simultaneously via intraperitoneal and intravenous injection to maximize the likelihood of establishing productive infections. Inoculation of simarteriviruses into unengrafted NSG-SGM3 and wildtype NOR/LtJ control mice did not result in viremia according to qRT-PCR detection of *N* RNA. However, in engrafted mice, productive infections were achieved with PBJV (all 5 mice), SHFV (4 of 6 mice), and SWBV-1 (2 of 7 mice); 1 engrafted mouse supported a brief infection with KRCV-1. Virus infections often persisted until the end of the study period (Figure 9); only 2 mice cleared virus RNA after viremia was detected.

## Discussion

Simarteriviruses commonly infect cercopithecoid monkeys throughout sub-Saharan Africa. Yet, lack of simarterivirus isolates impedes in vitro and in vivo investigations partly because of an extremely limited set of tools available for working with those viruses in a laboratory setting, including the lack of in vitro systems available for simarterivirus culture. We used a combination of increasingly sophisticated approaches (an immortalized grivet cell line, primary rhesus monkey PBMCs and splenocytes, crab-eating macaque iPSC-derived macrophages, and rhesus monkey leukocyte-engrafted laboratory mice) to create a straightforward toolset for isolating simarteriviruses. The combined effort resulted in isolation of the highly virulent, epizootic PBJV and successful infection of laboratory mice with diverse simarteriviruses. However, no single host system was capable of supporting robust replication of all 4 simarteriviruses tested in this study. Only SHFV replicated in immortalized MA-104 cells (Figure 1), and, although KRCV-1 did not replicate robustly in engrafted mice, SWBV-1 only replicated in engrafted mice (Figure 9). Our findings imply fundamental differences in tissue or host tropism among simarteriviruses. Nevertheless, we reveal a path for isolating other still-uncultured simarteriviruses and provide a system for evaluating medical countermeasures against simarteriviruses in vivo in highly characterized mouse models.

The 4 viruses isolated in this study are highly divergent from each other (≈50% genome identity) and represent only a portion of the known natural diversity of simarteriviruses (*11*). Functionally characterizing the many divergent simarteriviruses is critical to decipher the virus-host interactions that govern simarterivirus cross-species transmission. Although specific virus-host interactions remain largely unknown, the isolation systems developed in this study will expedite mechanistic studies of simarteriviruses in the laboratory to elucidate those interactions, to assess their zoonotic risk (*3*–*5*), and to develop candidate medical countermeasures.

Appendix Additional information for isolation of diverse simian arteriviruses causing hemorrhagic disease.
